# Malaysian public preferences and decision making for COVID-19 vaccination: A discrete choice experiment

**DOI:** 10.1016/j.lanwpc.2022.100534

**Published:** 2022-08-09

**Authors:** Hoon Shien Teh, Yuan Liang Woon, Chin Tho Leong, Nicholas Yee Liang Hing, Teresa Yong Sui Mien, Laurence S.J. Roope, Philip M. Clarke, Lee-Ling Lim, John Buckell

**Affiliations:** a Centre for Clinical Epidemiology, Institute for Clinical Research, National Institute of Health, Ministry of Health Malaysia, Persiaran Setia Murni, Setia Alam, 40170 Shah Alam, Selangor, Malaysia; b Institute for Health Behavioral Research, National Institute of Health, Ministry of Health Malaysia, Persiaran Setia Murni, Setia Alam, 40170 Shah Alam, Selangor, Malaysia; c Health Economics Research Centre, Nuffield Department of Population Health, University of Oxford, Oxford, United Kingdom; d NIHR Oxford Biomedical Research Centre, United Kingdom; e Department of Medicine, Faculty of Medicine, University of Malaya, Kuala Lumpur, Malaysia; f Department of Medicine and Therapeutics, The Chinese University of Hong Kong, Hong Kong SAR, China; g Asia Diabetes Foundation, Hong Kong SAR, China

**Keywords:** COVID-19, Vaccine, Discrete choice experiment, Uptake, Preference

## Abstract

**Background:**

Low vaccine uptake has the potential to seriously undermine COVID-19 vaccination programs, as very high coverage levels are likely to be needed for virus suppression to return life to normal. We aimed to determine the influence of vaccine attributes (including access costs) on COVID-19 vaccination preferences among the Malaysian public to improve national uptake.

**Methods:**

An online Discrete Choice Experiment (DCE) was conducted on a representative sample of 2028 Malaysians. Respondents were asked to make vaccination decisions in a series of hypothetical scenarios. A nested, mixed logit model was used to estimate the preferences for vaccination over vaccine refusal and for how those preferences varied between different sub-populations. The attributes were the risk of developing severe side effects of the vaccine, vaccine effectiveness, vaccine content, vaccination schedule, and distance from home to vaccination centre.

**Findings:**

Reported public uptake of COVID-19 vaccination was primarily influenced by the risk of developing severe side effects (b = −1·747, 95% CI = −2·269, -1·225), vaccine effectiveness (b = 3·061, 95% CI = 2·628, 3·494) and its Halal status (b = 3·722, 95% CI = 3·152, 4·292). Other factors such as appointment timing and travel distance to the vaccination centre also had an effect on vaccine uptake. There was substantial heterogeneity in preferences between different populations, particularly for age groups, ethnicity, regions, and underlying health conditions.

**Interpretation:**

Perceived effectiveness and side effects are likely to affect COVID-19 vaccine uptake in Malaysia. Halal content is critical to Malays’ vaccination choices. Reducing the physical distance to vaccination centres, particularly in rural areas where uptake is lower, is likely to improve uptake.

**Funding:**

Ministry of Health Research Grant from the Malaysian government [NIH/800-3/2/1 Jld.7(46), grant reference no: 57377 and warrant no: 91000776].

**Research in context**

**Evidence before this study**

A rapid review was done on PubMed, Cochrane library and Scopus to identify studies on the uptake of COVID-19 vaccination. A comprehensive list of search terms with the application of MeSH and text-word was used (supplemental document Table S1). This review included 37 relevant articles published from 2019 to 2020 in English. Narrative synthesis of these articles shortlisted 19 relevant characteristics that are potentially relevant to increase vaccination uptake. Expectedly, safety and efficacy are the most frequently discussed factors relating to COVID-19 vaccination uptake. Elsewhere, evidence also suggests that these factors, alongside the enabling environment and certain social influences, create a complex decision-making landscape for individuals. The relative strengths of these influences on vaccination decisions are not known, nor is how the strength of these influences vary among different population sub-populations.

**Added value of this study**

COVID-19 vaccination intentions in Malaysia were assessed using a series of choice tasks between vaccinations that varied according to several characteristics. In each choice task, respondents could either choose one of the two presented vaccinations, or not to be vaccinated. The experiment enabled the development of a behavioural model that provides policy-relevant evidence on the relative importance of key vaccination characteristics on vaccination uptake in Malaysia. It also investigated how different groups, including those for which uptake is lower, value those characteristics differently. Knowing these features can help the government with its vaccination strategy, particularly in groups with lower uptake. Our study was designed by engaging policymakers, and administered through the Malaysian Ministry of Health website. Public uptake of COVID-19 vaccination was primarily influenced by the risk of developing severe side effects, vaccine effectiveness, and its Halal status. Distance travelled to receive the vaccine was the next most important factor. Appointment timing was less important than the other factors.

**Implications of all the available evidence**

Though Malaysia has completed its initial vaccination protocol of fully vaccinating over 80% of the population, the emergence of more transmissible COVID-19 variants necessitates maintaining as high a level of vaccination as possible. Consistent with previous research, our study confirmed that higher perceived vaccine effectiveness and reduced perceived risk of side effects appear likely to improve vaccine uptake. The study also provides novel evidence that, in Malaysia, Halal vaccine content is critical to Malays’ COVID-19 vaccination choices. This suggests that communicating the absence of non-Halal ingredients in COVID-19 vaccines could improve uptake. The study also found that distance to vaccination centre influences vaccination choices. Reducing the physical distance to vaccination and associated access costs, particularly in rural areas where uptake is lower, could encourage vaccine uptake.

Alt-text: Unlabelled box

## Introduction

A key strategy for ending the COVID-19 pandemic is vaccination of the global population. The recent emergence and dominance of new variants is an indication that ongoing use of booster vaccinations may be needed.^1^ Guided by the scientific community, policymakers initially anticipated that herd immunity would require COVID-19 vaccination rates of up to 80% of the population.^2^ Therefore, following the authorization of COVID-19 vaccines, Malaysia's National Immunization Program aimed to ensure that at least 80% of adults received a vaccine by February 2022 to reduce COVID-19-related infections, hospitalizations, and deaths.^3^

Uptake has been high and this goal has now been met, despite the existence of a substantial subgroup of the population who are vaccine hesitant.^4^ However, with the emergence of more transmissible variants that are better equipped to evade existing vaccines, the threshold for herd immunity has not only increased substantially; it is probably unachievable.^5^^,^^6^ This, together with recent evidence on the need to maintain immunity via booster shots, means that the highest possible vaccination rates will be needed going forward.^7^^,^^8^

A WHO technical report highlighted that COVID-19 vaccination uptake is driven by behavioral factors including an enabling environment, social influences and motivation.^9^ These three drivers may interact and complicate the decision-making behind vaccination uptake. While COVID-19 vaccines are available free of charge in most countries, including Malaysia, it is important to recognize that vaccine uptake will be influenced by access costs, such as the time and travel required to attend the place of vaccination.^10^ Further policy responses required to increase vaccine uptake will depend on the key sources of hesitancy. So, for example, concern about side-effects could be addressed via information campaigns, while reducing access costs requires policies such as use of mobile vaccination clinics or travel subsidies for those living further away from vaccine centres.

Discrete Choice Experiments (DCE) have been widely used to examine preferences for non-emergency vaccination programs, such as Human Papilloma Virus (HPV) vaccinations and seasonal influenza vaccinations.^11^, ^12^, ^13^, ^14^, ^15^ In the context of COVID-19 vaccination, this method has been used to study vaccination preferences in several countries, such as China, France and Australia.^16^, ^17^, ^18^, ^19^ While these studies have addressed some aspects of vaccine hesitancy, currently none have directly looked at the effects of access costs, compared with other factors that may influence vaccine hesitancy.

This study aimed to investigate the important attributes that might influence decision-making regarding COVID-19 vaccination in Malaysia. An important aspect of access in Malaysia is rural access to health care, as previous research has shown that distance has an impact on uptake.^20^ In this multiethnic country, vaccine content could potentially be deemed unacceptable for religious reasons, so this factor is also considered in our study.^21^^,^^22^ We were also interested to know how preferences for COVID-19 vaccination vary with sociodemographic factors.

## Methods

**Figure 1:**
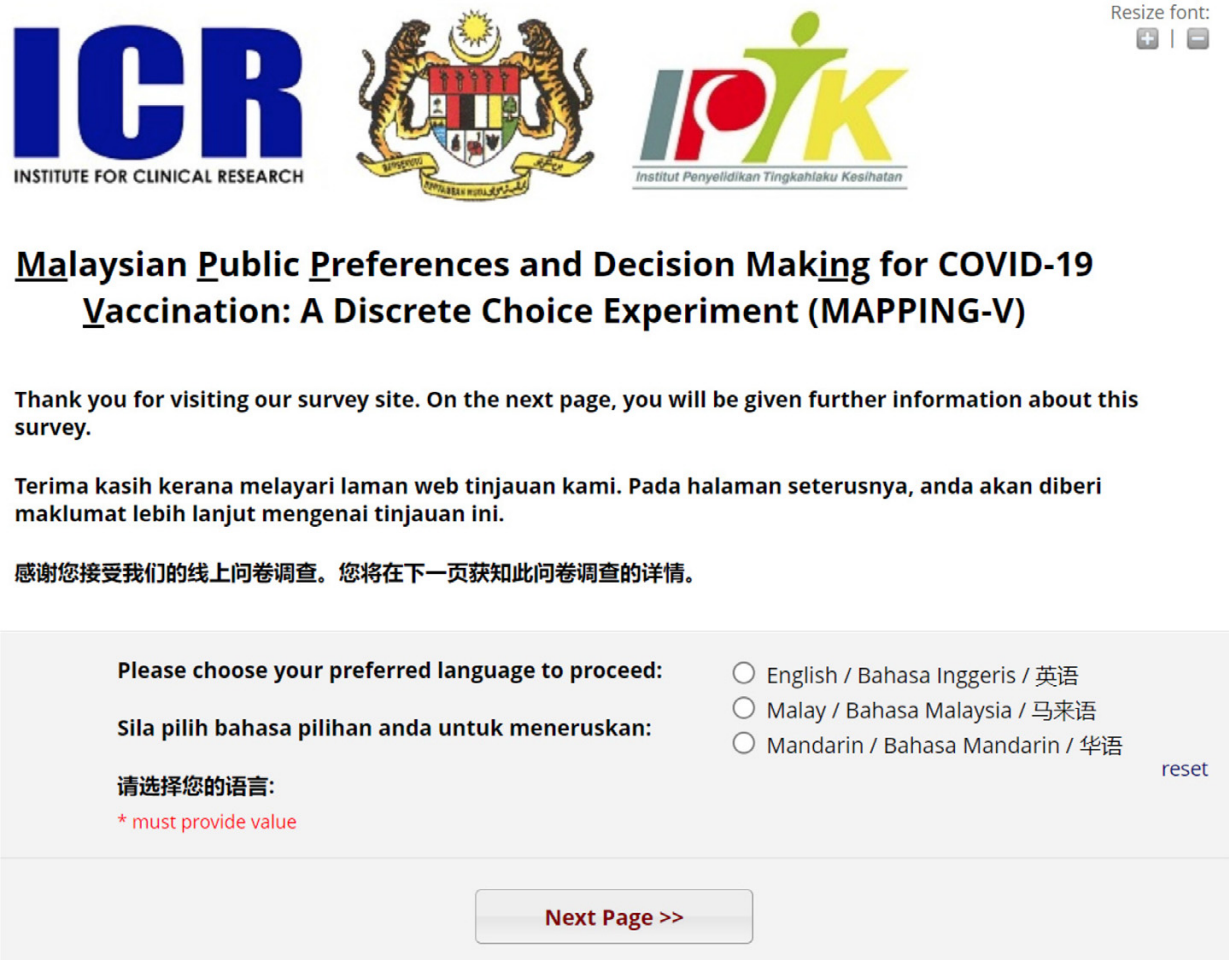
**Front page of the DCE survey**.

This DCE was conducted in March 2021, when the government had just initiated the National Immunization Program among healthcare workers. The inclusion criteria for the respondents were members of the Malaysian general public aged 18 years old and above. The survey was built on the online survey platform Research Electronic Data Capture (REDCap). An anonymized Self-Administered Questionnaire (SAQ) was circulated via government social media and by snowball sampling. The National Crisis Preparedness and Response Centre (CPRC) social media page was chosen as the dissemination platform as this was the official site for sharing COVID-19 related information from the Ministry of Health (MOH). This is important for incentive compatibility. In contrast to surveys administered by other entities (e.g., market research companies or even universities), respondents are likely to feel incentised to answer a survey administered by the official MOH truthfully, as the MOH could plausibly implement changes to the national vaccine program, based on survey results. The front page of the survey clearly indicated that it was from the MOH (Figure 1).

### DCE experimental design

We conducted a rapid review to identify the main attributes for vaccination uptake for COVID-19 routine vaccination. Articles were extracted from PubMed, Cochrane library and Scopus. The search terms used are listed in Table S1 Supplemental Materials. The searches were conducted from 11 November to 13 November 2020 and were restricted to studies published in the English language from the year 2019 onwards. The search strategy was developed with an emphasis on factors that influence decision making for COVID-19 vaccination uptake.

A team of 10 experts (five physicians from the MOH, two academics from teaching hospitals, two researchers with previous experience in vaccine-related studies, and one public health specialist) were then invited to rank the 19 shortlisted attributes. A focus group discussion with the Disease Control Division team from the MOH and two one-to-one interviews with public health specialists were also conducted to discuss which attributes they deemed potentially important from a policy perspective – in the sense that these attributes could potentially be used as policy levers.

We included five vaccine-related attributes in the final experimental design that were amenable to policy. These were the risk of developing severe side effects (1 in 100,000, 1 in 10,000, 1 in 1000 and 1 in 100), based on risks published in government guidance for the public^23^^,^^24^; vaccine effectiveness to reduce infection risk (less than 70%, 70–90% and more than 90%), based on clinical trials’ reported efficacy^25^, ^26^, ^27^, ^28^ and the Malaysian government guidelines^3^; vaccine content (non-Halal and no bovine, non-Halal and contains bovine, Halal and no bovine, Halal and contains bovine); vaccination schedule based on Malaysia facilities’ opening hours (during office hour at public facility, during office hour at private facility, out-of-office hour at public facility and out-of-office hour at private facility); and distance from home to vaccination centre (1km, 5km, 10km, 50km). Attribute levels were defined based on the characteristics of known vaccines to make the choices realistic to respondents. The attributes were dummy coded, though the choice of coding makes no difference to the relative attribute level estimates.^29^ Distance from home to vaccination centre was treated continuously to estimate the relative marginal utility of a 1-unit change in this distance*.* The attributes and levels are displayed in Table 1.

**Table 1:** Attributes and levels chosen for this experiment.

Attributes	Levels
Severe side effects	very low risk (1 in 100,000)
	low risk (1 in 10,000)
	moderate risk (1 in 1000)
	high risk (1 in 100)
Effectiveness	less than 70%
	70–90%
	above 90%
Vaccine content	non-Halal (no bovine)
	non-Halal (contains bovine)
	Halal (no bovine)
	Halal (contains bovine)
Vaccination schedule	office hour (8 am to 5 pm) at public facility
	office hour (8 am to 5 pm) at private facility
	out-of-office hour (after 5pm; weekends) at public facility
	out-of-office hour (after 5pm; weekends) at private facility
Distance from home to vaccination facilities	1 km
	5 km
	10 km
	50 km

Note: am= before midday; pm=after midday; km=kilometre.

A Bayesian d-optimal efficient design with uniformly distributed priors generated forty choice tasks spread across four blocks. Priors were obtained from a pilot study on 65 individuals before the full data collection. Each respondent was randomly assigned to one of the four blocks and responded to ten choice tasks; this helps to balance learning effects and respondent fatigue.^30^ To further reduce any learning effects, a warm-up task was given to each respondent prior to the set of ten experimental tasks. Each choice task comprised two vaccine alternatives and an opt-out option. In addition, a dominance test was included as the last question in all blocks to assess participants’ attention and understanding, where all levels in one option (vaccine A) were unambiguously superior to the other (vaccine B). Those respondents who chose vaccine B in this dominance test were excluded for analysis (89 respondents in total). Prior to data collection, a pilot study was conducted among 65 individuals to assess the understanding of the survey and to update the experimental design. The participants were asked to complete the survey through the same online platform. One-to-one interviews were then conducted to gather feedback. Subsequent amendments were made to the language, the levels, and the priors used in the experimental design. An example of the choice task is provided in Table 2.

**Table 2:** Example of choice task.

Characteristics of COVID-19 vaccine/vaccination	Vaccine A	Vaccine B
**Severe side effects that may require you to be hospitalized**	1 in 100 will have severe side effects	1 in 1000 will have severe side effects
**Effectiveness**	70–90%	Less than 70%
**Content**	· Non-Halal certified · No ingredients from cow (bovine)	· Halal certified · Contains ingredients from cow (bovine)
**Schedule (all COVID-19 vaccinations are free)**	During office hours (weekdays, 8 am-5 pm) in the public healthcare facilities	Out-of-office hours (after 5 pm, weekends/public holidays) in the public healthcare facilities
**Distance from home to vaccination facilities**	10 km	50 km
**I choose** **(Please tick one box only)**	☐ Vaccine A ☐ Vaccine B ☐ No vaccination	

Note: am= before midday; pm=after midday; km=kilometre.

### Survey instrument

The final survey consisted of three main sections as shown in Supplemental Materials. The first section presented the DCE. The subsequent section captured socio-demographic information of the respondents, such as sex, race, age, marital status, and household income. The final section included questions on risk perception, general views on vaccination and efforts to tackle the COVID-19 pandemic.

### Sample size

Based on the pilot data estimates, power calculations were conducted using the approach of de Bekker Grob et al. (2015).^31^ The minimum sample size based on parameter values obtained from the pilot study was given by:

(1) $$N\geq {\left[\left(\;Z_{1-\beta }+\;Z_{1-\alpha }\right)\sqrt{\Sigma_{\gamma k\;}}/\delta \right]}^{2},$$

where: Σ_γk_ = variance covariance matrix; 1 − β = power level (0.8); α  = confidence level (95%); δ  =  effect size. To be practical, we calculated the minimum sample size on the assumption that the effect size was larger than 0.1. The required sample size ranged from 78 to 1465 for different coefficients with a statistical power of 0.8 and α  =  95%. Therefore, the total sample size of 2028 was in excess of the minimum required sample size.

### Ethical review

This study was approved by the Malaysia Research Ethics Committee (MREC) with a study identification number of NMRR-20-2633-57377. Consent was obtained from all participants after they had gone through the participant information sheet and prior to answering the survey questions.

### Data analysis

Based on McFadden (1974), respondents were assumed to maximize their utility in making choices.^32^ In this formulation, the individual reconciles their vaccine/attribute preferences for each of the available alternatives and chooses that which maximizes their utility; that is, choosing their preferred vaccine or no vaccine option in the choice tasks. Respondents’ utility is a linearly-additive function of vaccine/attribute preferences and the vaccine-attribute combinations available in each task.

(2) $$U_{ni\;}=\;V_{ni}+\varepsilon_{ni}=\sum_{m}\beta_{m}\cdot x_{im}+\varepsilon_{ni},$$

where: *U*_*ni*_ is the utility for decision-maker *n* of option *i*, comprising deterministic and random utility;  *V_ni_* is the deterministic component of utility; ε_*ni*_ is the random component of utility; *x_im_* is the *m*th attribute-level of option *i*; and β_*m*_ is the *m*th preference parameter to be estimated. The deterministic component of utility comprises preferences for vaccination (versus no vaccination), vaccine attributes, and survey artefacts (left-to-right bias):

(3) $$\begin{array}{c} V_{ni}+\varepsilon_{ni}=\text{AS}C_{vaccine}+\text{AS}C_{left-to-right-bias}\\ +\beta 1*Side\;Effect_{low\;}+\beta 2*Side\;Effect_{moderate}+\beta 3*\\ Side\;Effect_{high}+\beta 4*Effectiveness_{70-90}\\ +\beta 5*Effectiveness_{90}+\beta 6*\\ Content_{non-Halal\;contains\;bovine}+\beta 7*Content_{Halal\;no\;bovine}\\ +\beta 8*Content_{Halal\;contains\;bovine}+\beta 9*\\ Schedule_{office\;hour,\;private}\\ +\beta 10*Schedule_{out\;of\;office\;hour,\;public}+\beta 11*\\ Schedule_{out\;of\;office\;hour,\;private}+\beta 12*\text{Distance}+\varepsilon_{ni}, \end{array}$$

where: ASC is an alternative-specific constant. ASC_vaccine_ captures the average preference for having a vaccine (versus not having a vaccine) that is not captured by the attributes. ASC_left − to − right − bias_ accounts for a tendency to choose options presented on the left more often than those presented on the right. Estimation was operationalized by assuming a type-I extreme value error distribution on the error term and estimating choice probabilities for each product with a multinomial logit (MNL) model.

(4) $$P_{ni}=\frac{\text{exp}\left(V_{ni}\right)}{\sum_{j=1}^{n}\text{exp}\left(V_{nj}\right)},$$

where *P_ni_* is the random utility model probability that respondent *n* chooses product *i* from choice set *J*.

Under the Independence of Irrelevant Alternatives (IIA) assumption of the MNL model, there is no correlation assumed between alternatives. This is behaviorally unlikely in the case of the two vaccine options, the choices of which are likely related. A nested logit model allows for correlation between alternatives, in this case specifying a nesting structure with a “vaccine” group (with the two vaccine options) and a “no vaccine” group (containing the opt-out). We then estimate the probability of being in a group as well as the probability of vaccine choice, conditional on being in the vaccine group.

(5) $$P_{ni,g}=P_{ni,g}\left(Group\right).\;P_{ni,g}\left(Vaccine|Group\right)=\frac{\text{exp}\left(\lambda_{g}I_{ng}\right)}{\sum_{l=1\ldots L}\text{exp}\left(\lambda_{l}I_{nl}\right)}\;.\;\frac{\text{exp}\left(V_{ni}/\lambda_{g}\right)}{\sum_{j=1\ldots J}\text{exp}\left(V_{nj}/\lambda_{g}\right)}$$

Where $I_{nk}=\ln\sum_{j\in g}\text{exp}\left(V_{nj}/\lambda_{g}\right)$, g is each group (vaccine or no vaccine) and $\lambda_{g}$ is a within-group correlation parameter to be estimated.^33^

Heterogeneous responses of vaccine choices to attributes were treated deterministically and randomly. For deterministic heterogeneity, the vaccination ASC and the attributes were interacted with individuals’ characteristics (age, sex, etc.). Random preference heterogeneity was modelled using mixing distributions.^33^ The parameters are then re-specified as a distribution with a mean and a standard deviation to be estimated. Combining the forms of heterogeneity, taking β_1_*Side effect_low_ as an example, we have,

(6) $$\beta_{1}=\mu_{1}+\gamma^{\prime }Z_{i}+\theta_{1},$$

where μ_1_ is the mean and $\theta_{1}∼N\left(0,\sigma_{\theta ,1}^{2}\right)$ is a random component capturing unobserved preference heterogeneity for attribute 1. Normal distributions were specified for all attributes, and 500 draws were taken using Modified Latin Hypercube Sampling.^34^ *Z_i_* are individual characteristics (age, sex, etc.) and γ are parameters measuring differential preferences by individual characteristics, to be estimated. All models were estimated using simulated maximum likelihood in the Apollo package for R.^35^

After estimation, we used the fitted model to make behavioral forecasts under different vaccine scenarios. As is standard, we used sample enumeration for forecasting.^33^ We took means of predicted probabilities, rather than using hit rates, as recommended by Hess and Palma.^35^ A post-stratification adjustment on the individual level data corrected the education imbalance in our sample (whilst also continuing to balance age, gender, and region). These weights were then applied to the base nested logit.

### Role of the funding source

This work was supported by MOH Research Grant (91000776). LSJR, PMC and JB are supported by the NIHR Oxford Biomedical Research Centre. The funders had no roles in study design, data collection, data analysis, interpretation, or writing of the paper.

## Results

### Respondent characteristics

A total of 2028 individuals were included in the study. Table 3 presents the respondents’ characteristics. The study sample was broadly representative of the national population in 2020, according to age groups, sex, ethnicity, household income and regions of residence.^36^ Participants were also evenly distributed across the groups in each block (Table S2 Supplemental material). General views of the public on risk perception, vaccination and efforts against the COVID-19 pandemic are described in Table S3-S6 Supplemental material. There was oversampling of respondents with higher education levels. Based on sensitivity analysis results (Appendix 2 Supplemental material), this did not appear to affect the nested logit model estimates. There were no appreciable differences in the weighted regression for other variables either.

**Table 3:** Respondents characteristics (*N*=2028).

Characteristics	*N*	%	National statistics (%)
Age groups*			
18-39	1061	52·3	36·9
40-59	763	37·6	21·7
60 and above	204	10·1	11·5
**Sex**			
Male	1031	50·8	51·5
Female	997	49·2	48·5
**Ethnicity**			
Malay	1130	55·7	69·6
Chinese	781	38·5	22·6
Indian	95	4·7	6·8
Others	22	1·1	1·0
**Household income**			
Low income	783	38·6	40·0
Middle income	769	37·9	40·0
High income	476	23·5	20·0
**Region of residence**			
Central	825	40·7	32·1
North	385	19·0	20·6
South	184	9·1	11·6
East	139	6·9	14·8
Borneo	495	24·4	20·9
**Education**			
No formal education	18	0·9	5·8
Primary education	18	0·9	18·5
Secondary education	205	10·1	51·0
Tertiary education	1787	88·2	24·7

Note: *N*= Number of respondents in this DCE survey.

⁎ The average age of the sample is 40.9 years old.

### Main effects from the mixed, nested logit model

**Figure 2:**
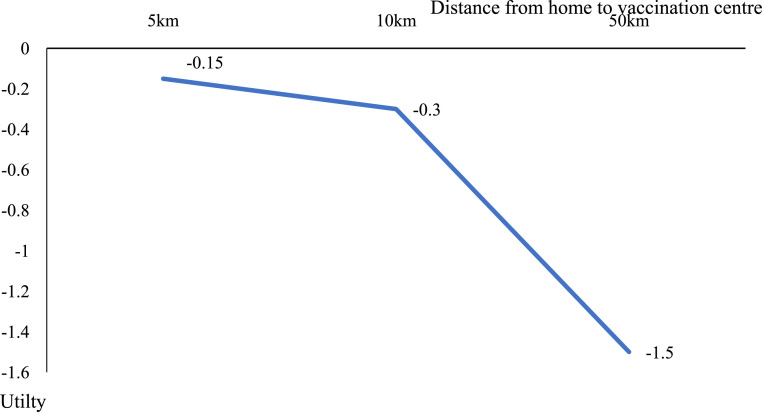
**Effect of distance on COVID-19 vaccination choice from the mixed nested logit model**.

Table 4 lists the main coefficients of the attributes from the mixed, nested logit model Eqs. (4)-((5) above). All attributes were significant except for bovine content and vaccination schedule at public or private facilities. Vaccine effectiveness and Halal content had a positive impact on utility. The risk of developing severe side effects, vaccination schedule during office hours, and distance from home to vaccination centre had negative impacts on utility. Halal content (b = 3·722, 95% CI = 3·152 to 4·292), effectiveness >90% (b = 3·061, 95% CI = 2·628 to 3·494) and high risk of side effects (b = −1·747, 95% CI = −2·269 to −1·225) of vaccines were the most important attributes influencing vaccination uptake. Distance travelled to receive the vaccine was the next most important factor (b = −0·03, 95% CI = −0·04 to −0·02). Further travel was a disincentive, but a distance of 50km did not offset gains from Halal, safety, or effectiveness (Figure 2). Vaccination during office hours was less important than the other factors (b = −0·18, 95% CI = −0·296 to −0·064).

**Table 4:** Main effects from the mixed nested logit model.

Attribute	Attribute level	b Coefficient (SE)	95% CI	
Vaccine content	Non-Halal	Reference	-	-
	Halal	3·722 (0·29)	3·152	4·291
Vaccine Effectiveness	Effectiveness 70%	Reference	-	-
	Effectiveness 70-90%	1·959 (0·14)	1·687	2·231
	Effectiveness >90%	3·061 (0·22)	2·628	3·494
Severe side effects of vaccine	Very low	Reference	-	-
	Low	−0·79(0·09)	−0·979	−0·601
	Moderate	−1·466(0·19)	−1·834	−1·098
	High	−1·747 (0·27)	−2·269	−1·224
Vaccination schedule	Non office hour	Reference	-	-
	Office hour	−0·18 (0·06)	−0·296	−0·064
Distance from home to vaccination facilities (continuous scale)		−0·03(0·01)	−0·040	−0·020

Note: SE: Standard error.

### Deterministic preference heterogeneity

Tables 5 reports the coefficients of the attribute levels in different populations from interactions. Results are presented for the total effect (main effect plus interaction) with confidence intervals adjusted accordingly. Overall, vaccine and attribute preferences varied by age group, ethnicity, region and underlying health conditions. Preferences for the distance to vaccination centre appeared not to vary across demographic characteristics.

**Table 5:** Deterministic heterogeneity: Interactions of attributes with demographics.

Attribute	Attribute levels	Sex				Age groups					
		Male		Female		Young		Middle aged		Elderly	
		Coefficient (SE)	LCB, UCB	Coefficient (SE)	LCB, UCB	Coefficient (SE)	LCB, UCB	Coefficient (SE)	LCB, UCB	Coefficient (SE)	LCB, UCB
Vaccine content	Non-Halal	Reference	-	Reference	-	Reference	-	Reference	-	Reference	-
	Halal	3·722	3·152, 4·292	4·604	3·970, 5·237	4·295	3·581, 5·008	3·722	3·152, 4·292	2·879	2·256, 3·502
Vaccine Effectiveness	Effectiveness 70%	Reference	-	Reference	-	Reference	-	Reference	-	Reference	-
	Effectiveness 70-90%	1·959	1·687, 2·231	1·959	1·687, 2·231	1·959	1·687, 2·231	1·959	1·687, 2·231	1·959	1·687, 2·231
	Effectiveness >90%	3·061	2·628, 3·494	3·061	2·628, 3·494	3·594	3·017, 4·172	3·061	2·628, 3·494	1·158	0·563, 1·752
Severe side effects of vaccine	Very low	Reference	-	Reference	-	Reference	-	Reference	-	Reference	-
	Low	-0·79	-0·979, -0·601	-0·79	-0·979, -0·601	-0·442	-0·719, -0·165	-0·79	-0·979, -0·601	-0·79	-0·979, -0·601
	Moderate	-1·466	-1·834, -1·098	-1·466	-1·834, -1·098	-0·747	-1·184, -0·311	-1·466	-1·834, -1·098	-1·466	-1·834, -1·098
	High	-1·747	-2·267, -1·225	-2·434	-2·971, -1·897	-1·747	-2·269, -1·225	-1·747	-2·267, -1·225	-1·747	-2·267, -1·225
Vaccination schedule	Non office hour	Reference	-	Reference	-	Reference	-	Reference	-	Reference	-
	Office hour	-0·18	-0·296, -0·064	-0·18	-0·296, -0·064	-0·18	-0·296, -0·064	-0·18	-0·296, -0·064	0·134	-0·214, 0·482
Distance from home to vaccination facilities (continuous scale)		-0·03	-0·040, -0·020	-0·03	-0·040, -0·020	-0·03	-0·040, -0·020	-0·03	-0·040, -0·020	-0·03	-0·040, -0·020

Attribute	Attribute levels	Presence of health conditions				Ethnicity					
		Yes		No		Malay		Chinese		Indian	
		Coefficient (SE)	LCB, UCB	Coefficient (SE)	LCB, UCB	Coefficient (SE)	LCB, UCB	Coefficient (SE)	LCB, UCB	Coefficient (SE)	LCB, UCB
Vaccine content	Non-Halal	Reference	-	Reference	-	Reference	-	Reference	-	Reference	-
	Halal	3·722	3·152, 4·292	3·722	3·152, 4·292	3·722	3·152, 4·292	-0·315	-0·705, 0·075	-0·541	-1·266, 0·185
Vaccine Effectiveness	Effectiveness 70%	Reference	-	Reference	-	Reference	-	Reference	-	Reference	-
	Effectiveness 70-90%	1·959	1·687, 2·231	1·959	1·687, 2·231	1·959	1·687, 2·231	2·514	2·154, 2·875	3·044	2·293-3·796
	Effectiveness >90%	3·061	2·628, 3·494	3·061	2·628, 3·494	3·061	2·628, 3·494	3·973	3·366, 4·580	5·011	3·830, 6·192
Severe side effects of vaccine	Very low	Reference	-	Reference	-	Reference	-	Reference	-	Reference	-
	Low	-0·79	-0·979, -0·601	-0·79	-0·979, -0·601	-0·79	-0·979, -0·601	-0·790	-0·979, -0·601	-0·790	-0·979, -0·601
	Moderate	-1·856	-2·314, -1·398	-1·466	-1·834, -1·098	-1·466	-1·834, -1·098	-2·099	-2·546, -1·653	-1·466	-1·834, -1·098
	High	-2·570	-3·231, -1·908	-1·747	-2·267, -1·225	-1·747	-2·267, -1·225	-2·836	-3·464, -2·209	-1·747	-2·267, -1·225
Vaccination schedule	Non office hour	Reference	-	Reference	-	Reference	-	Reference	-	Reference	-
	Office hour	0·108	-0·115, 0·331	-0·18	-0·296, -0·064	-0·18	-0·296, -0·064	-0·180	-0·296, -0·064	-0·180	-0·296, -0·064
Distance from home to vaccination facilities (continuous scale)		-0·03	-0·040, -0·020	-0·03	-0·040, -0·020	-0·03	-0·040, -0·020	-0·030	-0·040, -0·020	-0·014	-0·030, 0·002

Attribute	Attribute levels	Region of residence									
		Central		North		South		East		Borneo	
		Coefficient (SE)	LCB, UCB	Coefficient (SE)	LCB, UCB	Coefficient (SE)	LCB, UCB	Coefficient (SE)	LCB, UCB	Coefficient (SE)	LCB, UCB
Vaccine content	Non-Halal	Reference	-	Reference	-	Reference	-	Reference	-	Reference	-
	Halal	3·722	3·152, 4·292	3·722	3·152, 4·292	3·722	3·152, 4·292	3·722	3·152, 4·292	1·970	1·262, 2·678
Vaccine Effectiveness	Effectiveness 70%	Reference	-	Reference	-	Reference	-	Reference	-	Reference	-
	Effectiveness 70-90%	1·959	1·687, 2·231	1·959	1·687, 2·231	1·959	1·687, 2·231	1·959	1·687, 2·231	1·959	1·687, 2·231
	Effectiveness >90%	3·061	2·628, 3·494	3·061	2·628, 3·494	3·061	2·628, 3·494	3·061	2·628, 3·494	3·986	3·228, 4·743
Severe side effects of vaccine	Very low	Reference	-	Reference	-	Reference	-	Reference	-	Reference	-
	Low	-0·79	-0·979, -0·601	-0·790	-0·979, -0·601	-0·149	-0·635, 0·338	-0·79	-0·979, -0·601	-0·315	-0·773, 0·143
	Moderate	-1·466	-1·834, -1·098	-1·466	-1·834, -1·098	-0·707	-1·346, -0·067	-1·466	-1·834, -1·098	-0·690	-1·215, -0·165
	High	-1·747	-2·267, -1·225	-1·747	-2·267, -1·225	-0·642	-1·419, 0·135	-1·747	-2·267, -1·225	-0·823	-1·607, -0·039
Vaccination schedule	Non office hour	Reference	-	Reference	-	Reference	-	Reference	-	Reference	-
	Office hour	-0·180	-0·296, -0·064	-0·180	-0·296, -0·064	-0·180	-0·296, -0·064	-0·180	-0·296, -0·064	-0·180	-0·296, -0·064
Distance from home to vaccination facilities (continuous scale)		-0·030	-0·040, -0·020	-0·020	-0·031, -0·009	-0·020	-0·030, -0·005	-0·030	-0·040, -0·020	-0·030	-0·040, -0·020

Attribute	Attribute levels	Education level				Income level					
		Low		High		Low		Moderate		High	
		Coefficient (SE)	LCB, UCB	Coefficient (SE)	LCB, UCB	Coefficient (SE)	LCB, UCB	Coefficient (SE)	LCB, UCB	Coefficient (SE)	LCB, UCB
Vaccine content	Non-Halal	Reference	-	Reference	-	Reference	-	Reference	-	Reference	-
	Halal	3·722	3·152, 4·292	4·269	3·647, 4·891	3·722	3·152, 4·292	3·722	3·152, 4·292	3·722	3·152, 4·292
Vaccine Effectiveness	Effectiveness 70%	Reference	-	Reference	-	Reference	-	Reference	-	Reference	-
	Effectiveness 70-90%	1·959	1·687, 2·231	1·959	1·687, 2·231	1·959	1·687, 2·231	1·959	1·687, 2·231	1·959	1·687, 2·231
	Effectiveness >90%	3·061	2·628, 3·494	3·061	2·628, 3·494	3·061	2·628, 3·494	3·061	2·628, 3·494	3·061	2·628, 3·494
Severe side effects of vaccine	Very low	Reference	-	Reference	-	Reference	-	Reference	-	Reference	-
	Low	-0·79	-0·979, -0·601	-0·79	-0·979, -0·601	-0·79	-0·979, -0·601	-0·79	-0·979, -0·601	-0·79	-0·979, -0·601
	Moderate	-1·466	-1·834, -1·098	-2·082	-2·439, -1·726	-1·466	-1·834, -1·098	-1·466	-1·834, -1·098	-1·466	-1·834, -1·098
	High	-1·747	-2·267, -1·225	-2·919	-3·477, -2·361	-1·747	-2·267, -1·225	-1·747	-2·267, -1·225	-1·747	-2·267, -1·225
Vaccination schedule	Non office hour	Reference	-	Reference	-	Reference	-	Reference	-	Reference	-
	Office hour	-0·18	-0·296, -0·064	-0·18	-0·296, -0·064	-0·18	-0·296, -0·064	-0·18	-0·296, -0·064	-0·18	-0·296, -0·064
Distance from home to vaccination facilities (continuous scale)		-0·03	-0·040, -0·020	-0·03	-0·040, -0·020	-0·03	-0·040, -0·020	-0·03	-0·040, -0·020	-0·03	-0·040, -0·020

Note: SE= Standard error; LCB= Lower Confidence Bound; UCB= Upper Confidence Bound. If the utility values are the same, there was no preference heterogeneity detected across the groups.

Young adults (18–29 years old) (b = 3·594, 95% CI = 3·017 to 4·172) were more sensitive than middle-aged adults to effectiveness; older adults were less sensitive than middle-aged adults to effectiveness (b = 1·158, 95% CI = 0·563 to 1·752).

By ethnicity, Malays’ preferences for Halal content of the vaccines were the same as the average in the sample (b = 3·722, 95% CI = 3·152−4·292), but both Indian (b = −0·541, 95% CI = −1·266 to 0·185) and Chinese (b = −0·315, 95% CI = −0·705 to 0·075) ethnicities were indifferent to Halal content. Chinese respondents were more sensitive than Malay respondents to high risk of severe side effects (b = −2·836, 95% CI = −3·464 to −2·209) and effectiveness of vaccines (b = 3·973, 95% CI = 3·366 to 4·580). Indian respondents were more sensitive than Malay respondents to effectiveness higher than 70% (b = 3·044, 95% CI = 2·293 to 3·796) and effectiveness >90% (b = 5·011, 95% CI = 3·830 to 6·192). Respondents with existing health conditions exhibited a greater strength of preference for vaccines with less than moderate risk of developing severe side effects than those without existing health conditions (b = −1·856, 95% CI = −2·314 to −1·398 versus b = −1·466, 95% CI = −1·834 to −1·098).

In comparison to Central regions, respondents in Borneo and South regions were less sensitive to high risk of developing severe side effects (b = −0·823, b = −0·642 respectively). Respondents in Borneo also preferred a highly efficacious vaccine (b = 3·986, 95% CI = 3·228 to 4·743) compared to other regions.

### Forecast on vaccination uptake

**Figure 3:**
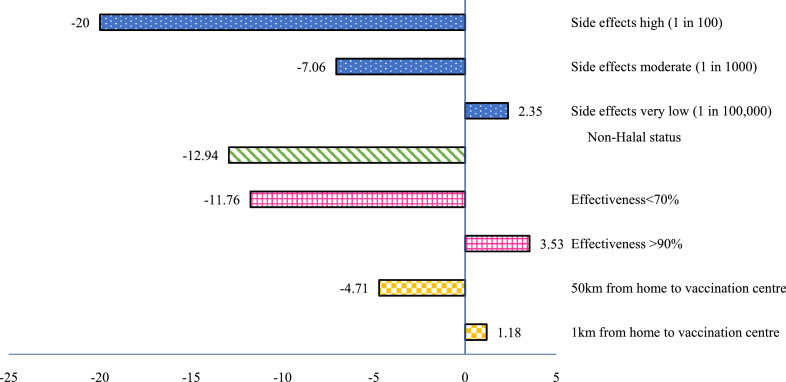
**Effects of changing the attribute levels on the probability of choosing a COVID-19 vaccination from base uptake of 85%**. The scenario from which these attribute levels were varied is: side effects low (1 in 10,000), Halal status, effectiveness of 70%-90%, and distance to vaccination center of 5km.

Figure 3 presents the change in vaccine uptake probabilities as vaccine attributes vary. We defined a base case vaccine to best replicate reality by setting: low risk of severe side effects (1 in 10,000), effectiveness 70–90%, Halal status and 10km distance from home to vaccination centre. Compared to the base case, very low risk of severe side effects, vaccine effectiveness of at least 90%, and a short distance between home and vaccination centre increased the probability of vaccination uptake. When the risk of severe side effects increased from low to high, the vaccination uptake probability decreased by around 20%. Non-Halal content of the vaccines decreased the vaccination uptake probability by around 13%. When the vaccine effectiveness was below 70%, the vaccination uptake probability decreased by around 12%. When the distance from home to the vaccination centre was as far as 50km, the vaccination uptake probability dropped by 5%.

## Discussion

Malaysia's National Immunization Program has exceeded its target of ensuring that at least 80% of adults received a vaccine by February 2022. The high vaccination levels are consistent with a variety of public surveys that have looked into acceptance levels for COVID-19 vaccination in Malaysia. The findings demonstrated encouraging acceptance rates with different magnitudes, ranging from 67% to 94% across different timelines.^37^, ^38^, ^39^ Rather than predicting uptake levels, the main purpose of our study was to disentangle the extent to which specific attributes of vaccination are likely to increase or reduce hesitancy, with a view to assessing which of these may be amenable to policy levers. Our study found that the risk of developing severe side effects, vaccine effectiveness and Halal content strongly influence vaccine preferences. The general Malaysian population is willing to accept an extremely low risk of developing severe side effects from COVID-19 vaccines (1 in 100,000). All else equal, a vaccine of such low risk would increase uptake by approximately 3% (Figure 3). The analyses also indicated that respondents preferred vaccines with at least 70% effectiveness. This study also sheds new light on another less addressed concern for COVID-19 vaccination, which was Halal content of the vaccines. Even though current COVID-19 vaccine candidates in Malaysia do not contain non-Halal ingredients, the hesitancy to accept could be due to common religious beliefs among the public. To a lesser extent, the other characteristics that could favor vaccination uptake were vaccination schedules during out-of-office hours and availability of vaccination centre within a 10km radius from home. The preference for out-of-office hours could be due to the working commitment of the population, which is mainly in the age group below 60 years old.

To the best of our knowledge, this study was the first in Malaysia to investigate public preferences for COVID-19 vaccination using a DCE. It used qualitative work, a pilot study (allowing our final models to be powered), and policymaker input to inform the design. Importantly, the study was administered by the government, giving the survey legitimacy and thus helping to elicit truthful responses. (Indeed, the findings have been shared with policymakers to help them fine tune their vaccination strategy). The sample was large and nationally representative according to most standard socio-demographic characteristics. It used complex models to capture the rich behaviors of individuals.

Limitations include potential selection bias due to online administration of the survey. Also, there was an oversampling of respondents with tertiary education compared to national statistics (88% vs 25%). In addition, common issues such as hypothetical bias and framing effects could have been present,^40^^,^^41^ though we submit that such biases are likely to be reduced in this study as the survey was administered by the government.

### Comparison with previous literature

Several studies in other countries have reported similar concerns on vaccine safety and effectiveness.^16^^,^^18^^,^^19^ Our findings were in line with another local vaccine acceptance survey conducted in early January 2021, in which the main reasons for people in Malaysia not accepting COVID-19 vaccines were the intention to wait for more data on vaccine safety and effectiveness.^38^ In regards to Halal content, our study concurs with a study by Wong et al. during the H1N1 outbreak in 2009. That study highlighted that whether a vaccine was Halal was the main factor that determined Malay participants’ decision to accept vaccination, whereas safety of the vaccine was the main factor that influenced vaccination decisions for Chinese and Indian participants.^42^

The expected vaccination uptake based on simulated scenarios in this study was consistent with the predicted COVID-19 vaccine hesitancy in France.^19^ In a study by Schwarzinger et al., the predicted hesitancy was found to be lowest for a COVID-19 vaccine with 90% efficacy and 1 in 100,000 risk of severe side effects (vaccine acceptance 61.3%). Our study captured a significant decrease in vaccination uptake for attribute levels which were inferior to the indicated reference level (i.e., low risk of severe side effects (1 in 10,000), effectiveness 70–90%, Halal status and 10km distance from home to vaccination centre).

This study also highlights the importance of travel distance as a possible barrier, particularly if the vaccination centre is more than 50km away. These findings were consistent with accessibility issues in the childhood immunization program in Malaysia. A local study highlighted that long travelling time of more than 30 min to an immunization health facility was a significant predictor of children not being immunized.^43^

### Implications

From Alpha, then Beta, and now Omicron, it is now clear that the closer we get to 100% full vaccination, likely supplemented with regular boosters, the lower the ongoing damage is likely to be to public health and the economy. This underscores the potential value of attempting to find policy levers to further reduce levels of COVID-19 vaccine hesitancy. After a year of COVID-19 vaccination in Malaysia, states on the east peninsular (Pahang, Terengganu and Kelantan) and one of the Borneo states (Sabah) had lower vaccination progress than the rest.^44^ It is possible that greater distances and access costs may be a factor in these largely rural states, which all have low population densities and high absolute poverty levels.^45^^,^^46^ However, these states also share other similarities, such as a high proportion of Malay population, so it is possible that there are other factors behind the differences – including a relatively high proportion of respondents with concerns over the Halal status of vaccines.

There was preference heterogeneity among the respondents in this study. Knowledge of the differences in these preferences could help in designing targeted communications. For example, our study showed that the elderly and those with health conditions had a higher probability of being vaccinated if they were offered vaccines with lower risk of severe side effects - even if effectiveness levels were only moderate. This group is the most sensitive to severe side effects. The elderly may be vulnerable to side effects if they have existing comorbidities or frailty, which may reduce their demand for a vaccine. The concern over Halal content was not surprising considering the majority of the Malaysian population are Malay Muslim. This was true for those vaccines during H1N1 outbreaks and other childhood vaccinations in Malaysia.^42^^,^^47^

As the Malaysia government procured five types of vaccines with different characteristics, this could hinder vaccination uptake. Certain populations could prefer one specific vaccine brand over the others due to the preferred characteristics. Our evidence allows health professionals to better understand which individuals would prefer which vaccines.

Time and travel costs can pose challenges for rural communities to access health care in Malaysia and elsewhere.^48^^,^^49^ Not only do isolated communities incur higher travel costs, there is a greater likelihood of needing to take time off work to visit a vaccination clinic. This may be compounded by the fact that such communities are likely to have lower incomes. Two strategies that could potentially lower access costs are mobile vaccination clinics, which would reduce travel distances, or some form of travel subsidy or incentive payment.

As expected, effectiveness and side effects were impactful on decisions to take vaccines against COVID-19. We provide new evidence that Halal content is critical to Malays’ vaccination choices, and that distance to vaccination centre influences uptake. In Malaysia, reducing the physical distance to vaccination, particularly in rural areas where uptake is lower, would encourage uptake.

## Contributors

HST: conceptualisation, data curation, formal analysis, funding acquisition, investigation, methodology, project administration, resources, software, validation, visualisation, writing – original draft; YLW: conceptualisation, funding acquisition, investigation, methodology, project administration, resources, supervision, visualisation, and writing – review & editing; CTL: methodology, project administration, resources, software, validation, visualisation, and writing – review & editing; NYLH: conceptualisation, methodology, project administration, validation, visualisation, and writing – review & editing; TYSM:conceptualisation, methodology, project administration, validation, visualisation, and writing – review & editing; LSJR: conceptualisation, methodology, supervision, validation, visualisation, and writing – review & editing; PMC: conceptualisation, methodology, supervision, and writing – review & editing; LLL: conceptualisation, methodology, and writing – review & editing; JB: conceptualisation, data curation, formal analysis, methodology, project administration, resources, software, supervision, validation, visualisation, and writing – review & editing.

## Data sharing statement

The datasets used and/or analyzed during the current study are available upon request to the corresponding author.

## Ethics committee approval

Ethical approval for this study was obtained from the Medical Research and Ethics Committee (MREC), Ministry of Health Malaysia. We have obtained permission from the Director General of Health Malaysia (Tan Sri Dato’ Seri Dr. Noor Hisham Abdullah) to publish this article.

## Declaration of interests

The authors declare that they have no conflicts of interests.

## Appendix Supplementary materials

Image, application 1

## References

[1] Dolgin E. (2021). Omicron is supercharging the COVID vaccine booster debate. Nature.

[2] Anderson RM, Vegvari C, Truscott J, Collyer BS. (2020). Challenges in creating herd immunity to SARS-CoV-2 infection by mass vaccination. Lancet.

[3] The special Committee for Ensuring Access to COVID-19 Vaccine Supply (JKJAV). National COVID-19 immunisation programme. 2021. https://www.vaksincovid.gov.my/pdf/National_COVID-19_Immunisation_Programme.pdf.

[4] Syed Alwi SAR, Rafidah E, Zurraini A, Juslina O, Brohi IB, Lukas S. (2021). A survey on COVID-19 vaccine acceptance and concern among Malaysians. BMC Public Health.

[5] Aschwanden C. (2021). Five reasons why COVID herd immunity is probably impossible. Nature.

[6] Hodgson D, Flasche S, Jit M (2021). The potential for vaccination-induced herd immunity against the SARS-CoV-2 B.1.1.7 variant. Eurosurveillance.

[7] Pouwels KB, Pritchard E, Matthews PC (2021). Effect of Delta variant on viral burden and vaccine effectiveness against new SARS-CoV-2 infections in the UK. Nat Med.

[8] Eyre DW, Taylor D, Purver M, et al. The impact of SARS-CoV-2 vaccination on Alpha & Delta variant transmission. *medRxiv* 2021: 2021.09.28.21264260.

[9] World Health Organization (WHO). Behavioural considerations for acceptance and uptake of COVID-19 vaccines: WHO technical advisory group on behavioural insights and sciences for health, meeting report.

[10] O'Donnell O (2007). Access to health care in developing countries: breaking down demand side barriers Acesso aos cuidados de saúde nos países em desenvolvimento: rompendo barreiras contra a demanda. Cad Saude Publica.

[11] Rubinstein H, Marcu A, Yardley L, Michie S. (2015). Public preferences for vaccination and antiviral medicines under different pandemic flu outbreak scenarios. BMC Public Health.

[12] Ledent E, Gabutti G, de Bekker-Grob EW (2019). Attributes influencing parental decision-making to receive the Tdap vaccine to reduce the risk of pertussis transmission to their newborn–outcome of a cross-sectional conjoint experiment in Spain and Italy. Hum Vaccines Immunother.

[13] Hoogink J, Verelst F, Kessels R (2020). Preferential differences in vaccination decision-making for oneself or one's child in the Netherlands: a discrete choice experiment. BMC Public Health.

[14] Wong CKH, Man KKC, Ip P, Kwan M, McGhee SM. (2018). Mothers’ preferences and willingness to pay for human papillomavirus vaccination for their daughters: a discrete choice experiment in Hong Kong. Value Heal.

[15] Arbiol J, Yabe M, Nomura H, Borja M, Gloriani N, Yoshida SI. (2015). Using discrete choice modeling to evaluate the preferences and willingness to pay for leptospirosis vaccine. Hum Vaccines Immunother.

[16] Leng A, Maitland E, Wang S, Nicholas S, Liu R, Wang J. (2020). Individual preferences for COVID-19 vaccination in China. Vaccine.

[17] Dong D, Xu RH, Wong EL, et al. Public preference for COVID-19 vaccines in China: a discrete choice experiment. *Health Expect*. 2020; 23:1543–1578. 10.1111/hex.13140 PMC7752198 33022806

[18] Borriello A, Master D, Pellegrini A, Rose JM. (2020). Preferences for a COVID-19 vaccine in Australia. Vaccine.

[19] Schwarzinger M, Watson V, Arwidson P, Alla F, Luchini S (2021). COVID-19 vaccine hesitancy in a representative working-age population in France: a survey experiment based on vaccine characteristics. Lancet Public Heal.

[20] Mahmud A, Aljunid SM. (2018). Availability and accessibility of subsidized mammogram screening program in peninsular Malaysia: a preliminary study using travel impedance approach. PLoS One.

[21] Rumetta J, Abdul-Hadi H, Lee YK. (2020). A qualitative study on parents’ reasons and recommendations for childhood vaccination refusal in Malaysia. J Infect Public Health.

[22] Elkalmi RM, Jamshed SQ, Suhaimi AM. (2021). Discrepancies and similarities in attitudes, beliefs, and familiarity with vaccination between religious studies and science students in Malaysia: a comparison study. J Relig Health.

[23] National Center for Immunization and Respiratory Diseases. Pfizer-BioNTech COVID-19 vaccine reactions & adverse events. https://www.cdc.gov/vaccines/covid-19/info-by-product/pfizer/. Systemic Reactions (82.8%25 vs 70.6%25). Accessed 6 May 2022.

[24] Information for UK recipients on COVID-19 Vaccine AstraZeneca (Regulation 174).https://www.gov.uk/government/publications/regulatory-approval-of-covid-19-vaccine-astrazeneca/information-for-uk-recipients-on-covid-19-vaccine-astrazeneca. Accessed 6 May 2022.

[25] Polack FP, Thomas SJ, Kitchin N (2020). Safety and efficacy of the BNT162b2 mRNA Covid-19 vaccine. N Engl J Med.

[26] Lopez Bernal J, Andrews N, Gower C (2021). Effectiveness of Covid-19 vaccines against the B.1.617.2 (Delta) variant. N Engl J Med.

[27] Sheikh A, McMenamin J, Taylor B, Robertson C. (2021). SARS-CoV-2 Delta VOC in Scotland: demographics, risk of hospital admission, and vaccine effectiveness. Lancet.

[28] Voysey M, Clemens SAC, Madhi SA (2021). Safety and efficacy of the ChAdOx1 nCoV-19 vaccine (AZD1222) against SARS-CoV-2: an interim analysis of four randomised controlled trials in Brazil, South Africa, and the UK. Lancet.

[29] Daly A, Dekker T, Hess S. (2016). Dummy coding vs effects coding for categorical variables: clarifications and extensions. J Choice Model.

[30] Hess S, Hensher DA, Daly A. (2012). Not bored yet—revisiting respondent fatigue in stated choice experiments. Transp Res Part A Policy Pract.

[31] de Bekker-Grob EW, Donkers B, Jonker MF, Stolk EA. (2015). Sample size requirements for discrete-choice experiments in healthcare: a practical guide. Patient.

[32] McFadden D. Frontiers in econometrics, chapter conditional logit analysis of qualitative choice behavior. 1974: 105–42.

[33] Train Kenneth E. (2009).

[34] Hess S, Train K, Polak JW (2006). On the use of a Modified Latin Hypercube Sampling (MLHS) method in the estimation of a Mixed Logit Model for vehicle choice. Transp Res Part B.

[35] Hess S, Palma D. (2019). Apollo: a flexible, powerful and customisable freeware package for choice model estimation and application. J Choice Model.

[36] Department of Statistics Malaysia. Current population estimates, Malaysia 2020. 2020.

[37] Malaysian Red Crescent. The COVID-19: survey perception survey. https://twitter.com/KKMPutrajaya/status/1344580831206023169?s = 20. Accessed 2 June 2021.

[38] Vaghefi N, Chin PN, Ch'ng KS. Survey on attitudes to Covid-19 vaccination in Penang. 2021.https://penanginstitute.org/publications/issues/survey-on-attitudes-to-covid-19-vaccination-in-penang/. Accessed 2 June 2021.

[39] Wong LP, Alias H, Wong PF, Lee HY, AbuBakar S. (2020). The use of the health belief model to assess predictors of intent to receive the COVID-19 vaccine and willingness to pay. Hum Vaccines Immunother.

[40] Harrison M, Rigby D, Vass C, Flynn T, Louviere J, Payne K. (2014). Risk as an attribute in discrete choice experiments: A systematic review of the literature. Patient.

[41] Howard K SG (2009). Does attribute framing in discrete choice experiments influence willingness to pay? Results from a discrete choice experiment in screening for colorectal cancer. Value Heal.

[42] Wong LP, Sam IC. (2010). Factors influencing the uptake of 2009 H1N1 influenza vaccine in a multiethnic Asian population. Vaccine.

[43] Krishna D, Mohd Zulkefli NA, Md Said S, Mahmud A (2019). Sociodemographic and health care factors in determining immunization defaulters among preschool children in Petaling District, Selangor: a cross-sectional study in Malaysia. BMC Public Health.

[44] Vaccinations in Malaysia. https://covidnow.moh.gov.my/vaccinations/. Accessed 24 January 2022.

[45] Department of Statistics Malaysia. Current population estimates, Malaysia 2021. 2021.

[46] Department of Statistics Malaysia (2020).

[47] Khoo YSK, Ghani AA, Navamukundan AA, Jahis R, Gamil A. (2020). Unique product quality considerations in vaccine development, registration and new program implementation in Malaysia. Hum Vaccines Immunother.

[48] Falcon DJ (2019). The health care gap in rural Malaysia. Perspect Bus Econ.

[49] Moola S, Gudi N, Nambiar D (2021). A rapid review of evidence on the determinants of and strategies for COVID-19 vaccine acceptance in low- and middle-income countries. J Glob Health.

