# Supplementary material for: Malaysian public preferences and decision making for COVID-19 vaccination: A discrete choice experiment
Source: Lancet Reg Health West Pac. 2022 Aug 9;27:100534. doi: 10.1016/j.lanwpc.2022.100534 (PMC9359905; doi:10.1016/j.lanwpc.2022.100534)
Supplement: Supplementary file 1 [file mmc1.docx]

Contents

**Appendix 1**

[**Table S1 Search terms used in rapid review** 2](#_Toc102728442)

[**Table S2 Respondents ’demographics by blocks** 3](#_Toc102728443)

[**Table S3 Risk perception of public** 4](#_Toc102728444)

[**Table S4 General views on vaccine/vaccinations** 4](#_Toc102728445)

[**Table S5 Efforts against the COVID-19 pandemic** 4](#_Toc102728446)

[**Table S6 Information reliability** 4](#_Toc102728447)

[**Appendix 2: Sensitivity analysis** 5](#_Toc102728448)

[**Appendix 3: Survey Instrument** 6](#_Toc102728449)

# **Table S1 Search terms used in rapid review**

| Coronavirus [MeSH Terms] | AND | Vaccination [MeSH Terms] | AND | base, knowledge [MeSH Terms] |
| --- | --- | --- | --- | --- |
| severe acute respiratory syndrome virus [MeSH Terms] |  | active immunization [MeSH Terms] |  | adolescent behavior [MeSH Terms] |
| Coronaviridae [MeSH Terms] |  | acquired immunity [MeSH Terms] |  | crisis intervention [MeSH Terms] |
| covid*[Text Word] |  | active immunity [MeSH Terms] |  | decision making [MeSH Terms] |
| SARS*[Text Word] |  | prevention and control [MeSH Terms] |  | behavior, choice [MeSH Terms] |
| Ncov [Text Word] |  | vaccin*[Text Word] |  | behaviors, choice [MeSH Terms] |
| novel cov[Text Word] |  | immuni?ation[Text Word] |  | acceptability of health care [MeSH Terms] |
| 2019-nCoV [Text Word] |  | inoculat*[Text Word] |  | acceptability of healthcare [MeSH Terms] |
| corona*[Text Word |  |  |  | Refusal [MeSH Terms] |
|  |  |  |  | analyses, policy [MeSH Terms] |
|  |  |  |  | analysis, policy [MeSH Terms] |
|  |  |  |  | Attitude [MeSH Terms] |
|  |  |  |  | Belief [MeSH Terms] |
|  |  |  |  | Beliefs [MeSH Terms] |
|  |  |  |  | Intention [MeSH Terms] |
|  |  |  |  | behavio*[Text Word] |
|  |  |  |  | Uptake [Text Word] |
|  |  |  |  | intervention*[Text Word] |
|  |  |  |  | choice*[Text Word] |
|  |  |  |  | Knowledge [Text Word |
|  |  |  |  | accept*[Text Word] |
|  |  |  |  | refus*[Text Word] |
|  |  |  |  | polic*[Text Word] |
|  |  |  |  | determinant*[Text Word] |
|  |  |  |  | attitude*[Text Word] |
|  |  |  |  | belief*[Text Word] |
|  |  |  |  | intent*[Text Word] |
|  |  |  |  | decision*[Text Word] |

# **Table S2 Respondents ’demographics by blocks**

| **Characteristics** | **Block 1, n (%)** | **Block 2, n (%)** | **Block 3, n (%)** | **Block 4, n (%)** |
| --- | --- | --- | --- | --- |
| **Age groups** | | |  |  |
| 18-39 | 281 (55.4) | 275 (54.2) | 244 (48.1) | 262 (51.5) |
| 40-59 | 179 (35.3) | 173 (34.1) | 213 (42.0) | 198 (39.1) |
| 60 and above | 47 (9.3) | 59 (11.6) | 50 (9.9) | 48 (9.5) |
| **Gender** | | |  |  |
| Male | 252 (49.7) | 255 (50.3) | 267 (52.7) | 257 (50.7) |
| Female | 255 (50.3) | 252 (49.7) | 240 (47.3) | 250 (49.3) |
| **Ethnicity** | | |  |  |
| Bumiputera | 297 (58.6) | 270 (53.3) | 274 (54.0) | 289 (57.0) |
| Chinese | 183 (36.1) | 217 (42.8) | 189 (37.3) | 192 (37.9) |
| Indian | 22 (4.3) | 16 (3.2) | 38 (7.5) | 19 (3.8) |
| Others | 5 (1.0) | 4 (0.8) | 6 (1.2) | 7 (1.4) |
| **Household income** | | |  |  |
| Low income | 209 (41.2) | 182 (35.9) | 191 (37.7) | 201 (39.6) |
| Middle income | 195 (38.5) | 205 (40.4) | 177 (34.9) | 192 (37.9) |
| High income | 103 (20.3) | 120 (23.7) | 139 (27.4) | 114 (22.5) |
| **Staying regions** | | |  |  |
| Central | 186 (36.7) | 221 (43.6) | 215 (42.4) | 203 (40.0) |
| North | 108 (21.3) | 92 (18.2) | 91 (18.0) | 94 (18.5) |
| South | 43 (8.5) | 42 (8.3) | 48 (9.5) | 51 (10.1) |
| East | 35 (6.9) | 30 (5.9) | 39 (7.7) | 35 (6.9) |
| Borneo | 135 (26.6) | 122 (24.1) | 114 (22.5) | 124 (24.5) |
| **Education** | | | |  |
| No formal education | 6 (1.2) | 4 (0.8) | 3 (0.6) | 5 (1.0) |
| Primary education | 6 (1.2) | 2 (0.4) | 5 (1.0) | 5 (1.0) |
| Secondary education | 52 (10.3) | 46 (9.1) | 60 (11.8) | 47 (9.3) |
| Tertiary education | 311 (61.3) | 455 (89.8) | 439 (86.6) | 450 (88.8) |

# **Table S3 Risk perception of public**

| **Questions** | **Low, n (%)** | **Medium, n (%)** | **High, n (%)** |
| --- | --- | --- | --- |
| In your opinion, how risky are you to get infected by COVID-19 in the next 6 months? | 1019 (50.2) | 791 (39.0) | 218 (10.7) |
| If you were infected with COVID-19, how serious would it affect your health? | 666 (32.9) | 855 (42.3) | 501 (24.8) |

# **Table S4 General views on vaccine/vaccinations**

| **Questions** | **Yes, n (%)** | **No, n (%)** |
| --- | --- | --- |
| Do you believe that vaccines can protect us from serious diseases (e.g. measles, polio, smallpox, Tuberculosis (TB), etc.)? | 1818 (89.6) | 210 (10.4) |
| Have you ever been reluctant to get a vaccination in the past? | 323 (15.9) | 1705 (84.1) |
| Have you ever refused a vaccination in the past? | 152 (7.5) | 1876 (92.5) |
| Would you encourage your immediate family members (children, spouses etc.) to be vaccinated for COVID-19? | 1893 (93.3) | 135 (6.7) |
| Would you encourage your friends to be vaccinated for COVID-19? | 1899 (93.6) | 129 (6.4) |

# **Table S5 Efforts against the COVID-19 pandemic**

| **Questions** | **Disagree, n (%)** | **Not sure, n (%)** | **Agree, n (%)** |
| --- | --- | --- | --- |
| Overall, I trust the Government for the management of COVID-19. | 336 (16.6) | 326 (16.1) | 1366 (67.4) |
| The government takes into consideration experts’ advice (e.g., epidemiologists, virologists, pediatricians, psychologists, etc.) to manage the current COVID-19 situation. | 296 (14.6) | 372 (18.3) | 1360 (67.1) |
| The government is allocating enough resources to enforce the adopted measures and guidelines to manage the current COVID- 19 situation. | 463 (22.8) | 426 (21.0) | 1139 (56.2) |

# **Table S6 Information reliability**

| **Questions** | **Not reliable, n (%)** | **Not sure, n (%)** | **Reliable, n (%)** |
| --- | --- | --- | --- |
| Media (TV, Newspapers, Radio, etc.) | 156 (7.7) | 344 (17.0) | 1528 (75.4) |
| Official Government Websites (e.g., Ministry of Health, National Government) | 208 (10.3) | 185 (9.1) | 1635 (80.6) |
| Social Media Platform (e.g. WhatsApp, Facebook) | 740 (36.5) | 786 (38.8) | 502 (24.8) |
| Doctors | 42 (2.1) | 325 (16.0) | 1661 (81.9) |
| Friend, Relatives and Acquaintances | 571 (28.2) | 1060 (52.3) | 397 (19.6) |
| Scientists | 49 (2.4) | 496 (24.5) | 1483 (73.1) |

# **Appendix 2: Sensitivity analysis**

Unweighted model


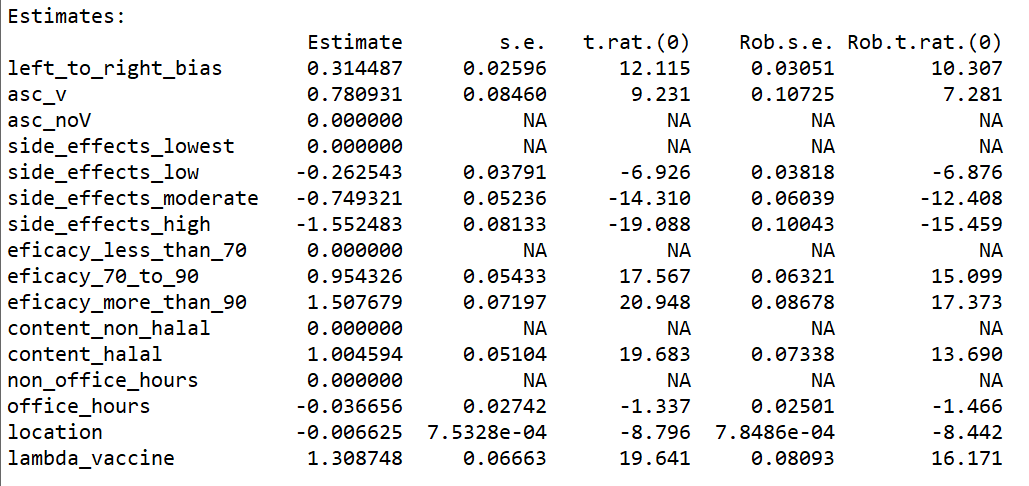


Weighted model with post-stratification


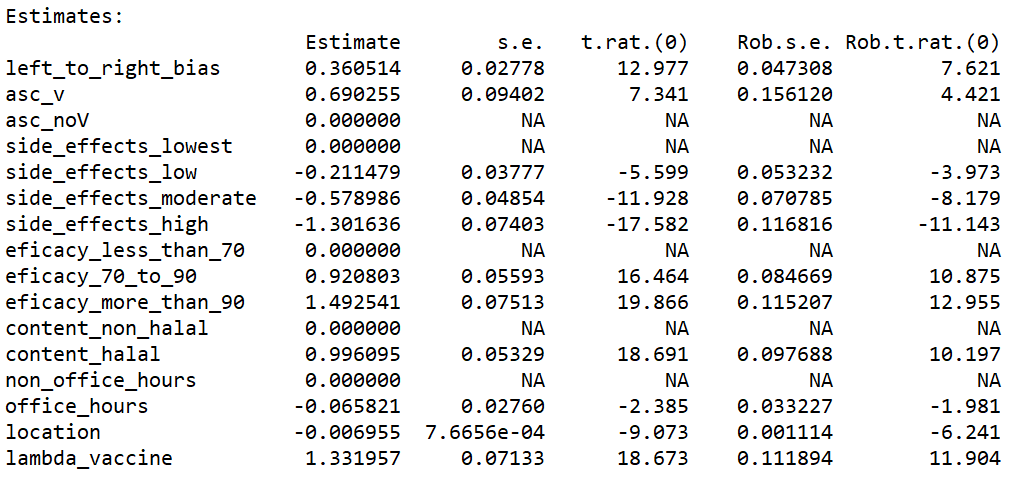


# **Appendix 3: Survey Instrument**

**Malaysian Public Preferences and Decision Making for COVID-19 Vaccination: A Discrete Choice Experiment (MAPPING-V)**

**PAGE 1:**

**Thank you for visiting our survey site. On the next page, you will be given further information about this survey. Please choose your preferred language to proceed.**

**English**

**Bahasa Malaysia**

**Mandarin**

**PAGE 2 （Eligibility questions）:**

**For verification purposes, please fill in the following information:**

| Q1. | Date of Birth (dd/mm/yyyy) | _ _ / _ _ / _ _ _ _ |
| --- | --- | --- |
| Q2. | Are you Malaysian? | Yes  No |

**Thank you! You are eligible to participate in this survey. Please click “NEXT PAGE” to continue to next section.**

**OR**

**Sorry, you are not eligible to participate in this survey. Thank you and no data will be collected.**

**PAGE 3:**

**This study is conducted and coordinated by the Institute for Clinical Research (ICR) and Institute for Health Behavioural Research (IHBR), Ministry of Health Malaysia. This study aims to understand the determinants of Malaysian public’s preferences for COVID-19 vaccination. The answers provided by you and other participants will help us understand vaccination related choices of Malaysian adults aged 18 and above. In the following pages, you will be asked to respond to a series of questions about your background and vaccination preferences.**

**Your participation in this study is completely voluntary. Your identity will remain anonymous as no personal identifiers will be collected in this study. If you decided to participate, you may change your mind and stop at any time. If you decide not to participate, there will not be any penalties. This survey will take at least 20 minutes to complete.**

**You may not copy, reproduce, distribute, publish, display, modify, create derivative works, transmit, or in any way exploit any such content, nor may you distribute any part of this content over any network, sell or offer it for sale, or use such content to construct any kind of database. Copying or storing any content is prohibited without prior written permission from the Institute for Clinical Research and Institute for Health Behavioural Research.**

**More detailed information of this study is attached in the link provided below. Please kindly read it carefully before you decide to participate.**

**By selecting “I AGREE AND CONSENT TO PARTICIPATE”, it means:**

- **You have read and understood the information provided; AND**
- **You agree to the terms as described; AND**
- **You are 18 years and above**

**I agree and consent to participate**

**I do not consent to participate**

**SECTION 1: CHOICE TASKS**

On each of the following pages, you will be given **11 scenarios related to Vaccine A and Vaccine B**.

Each scenario contains different vaccine / vaccination characteristics from different levels:

a) Severe side effects of the vaccine

b) Effectiveness of vaccines

c) Vaccine content

d) Vaccination schedule

e) Distance from home to vaccination facilities

Please **READ CAREFULLY** each feature in the given scenario before making your choice of vaccine / vaccination.

You can only select **1 answer** for each scenario, either:

1) Vaccine A (I am willing to receive vaccine A) or

2) Vaccine B (I am willing to receive vaccine B) or

3) Without Vaccination (I am not willing to receive both vaccines)

To begin, please complete the following **warm-up task.**

Based on the vaccine / vaccination characteristics below, what is your choice?

| **Characteristics of COVID-19 vaccine/vaccination** | **Vaccine A** | **Vaccine B** |
| --- | --- | --- |
| **Severe side effects that may require you to be hospitalized** | 1 in 100 will have severe side effects | 1 in 1,000 will have severe side effects |
| **Effectiveness** | **70-** **90%** | **Less than 70%** |
| **Content** | - **Non-Halal certified** - **No** ingredients from cow (bovine) | - **Halal certified** - **Contains** ingredients from cow (bovine) |
| **Schedule (all COVID-19 vaccinations are free)** | During **office hours (weekdays, 8am-5pm)** in the **public** healthcare facilities | **Out-of-office hours (after 5pm, weekends/public holidays)** in the **public** healthcare facilities |
| **Distance from home to vaccination facilities** | **10 km** | **50 km** |
| **I choose**  (please tick one box only) | **Vaccine A**  **Vaccine B**  **No vaccination** | |

**CONFIRMATION**

In this practice, you chose Vaccine A.

This vaccine is

- 1 in 100 will have severe side effects
- 70-90% effective
- Non-Halal certified with no ingredients from cow
- Free vaccination during office hours (weekdays, 8am-5pm) in the public healthcare facilities
- You have to travel 10km from home to get it.

**Is this your choice?**

Yes

No, thank you

**OR**

In this practice, you chose Vaccine B.

This vaccine is

- 1 in 1000 will have severe side effects
- Less than 70% effective
- Halal certified with ingredients from cow
- Free vaccination out-of-office hours (after 5pm, weekends/public holidays) in the public healthcare facilities
- You have to travel 50km from home to get it.

**Is this your choice?**

Yes

No, thank you

**OR**

In this practice, you chose No Vaccination

**Is this your choice?**

Yes

No, thank you

**Scenario 1 of 11:** Based on the vaccine / vaccination characteristics below, what is your choice?

| **Characteristics of COVID-19 vaccine/vaccination** | **Vaccine A** | **Vaccine B** |
| --- | --- | --- |
| **Severe side effects that may require you to be hospitalized** | 1 in 1,000 will have severe side effects | 1 in 10,000 will have severe side effects |
| **Effectiveness** | **70-** **90%** | **Above 90%** |
| **Content** | - **Halal certified** - **No** ingredients from cow (bovine) | - **Non-Halal certified** - **Contains** ingredients from cow (bovine) |
| **Schedule (all COVID-19 vaccinations are free)** | During **office hours (weekdays, 8am-5pm)** in the **public** healthcare facilities | **Out-of-office hours (after 5pm, weekends/public holidays)** in the **public** healthcare facilities |
| **Distance from home to vaccination facilities** | **1 km** | **50 km** |
| **I choose**  (please tick one box only) | **Vaccine A**  **Vaccine B**  **No vaccination** | |

**Scenario 2 of 11:** Based on the vaccine / vaccination characteristics below, what is your choice?

| **Characteristics of COVID-19 vaccine/vaccination** | **Vaccine A** | **Vaccine B** |
| --- | --- | --- |
| **Severe side effects that may require you to be hospitalized** | 1 in 1,000 will have severe side effects | 1 in 10,000 will have severe side effects |
| **Effectiveness** | **Less than 70%** | **Above 90%** |
| **Content** | - **Non-Halal certified** - **No** ingredients from cow (bovine) | - **Halal certified** - **No** ingredients from cow (bovine) |
| **Schedule (all COVID-19 vaccinations are free)** | **Out-of-office hours (after 5pm, weekends/public holidays)** in the **private** healthcare facilities | During **office hours (weekdays, 8am-5pm)** in the **public** healthcare facilities |
| **Distance from home to vaccination facilities** | **10 km** | **5 km** |
| **I choose**  (please tick one box only) | **Vaccine A**  **Vaccine B**  **No vaccination** | |

**Scenario 3 of 11:** Based on the vaccine / vaccination characteristics below, what is your choice?

| **Characteristics of COVID-19 vaccine/vaccination** | **Vaccine A** | **Vaccine B** |
| --- | --- | --- |
| **Severe side effects that may require you to be hospitalized** | 1 in 1,000 will have severe side effects | 1 in 10,000 will have severe side effects |
| **Effectiveness** | **70-90%** | **Less than 70%** |
| **Content** | - **Halal certified** - **No** ingredients from cow (bovine) | - **Halal certified** - **Contains** ingredients from cow (bovine) |
| **Schedule (all COVID-19 vaccinations are free)** | **Out-of-office hours (after 5pm, weekends/public holidays)** in the **public** healthcare facilities | During **office hours (weekdays, 8am-5pm)** in the **public** healthcare facilities |
| **Distance from home to vaccination facilities** | **1 km** | **50 km** |
| **I choose**  (please tick one box only) | **Vaccine A**  **Vaccine B**  **No vaccination** | |

**Scenario 4 of 11:** Based on the vaccine / vaccination characteristics below, what is your choice?

| **Characteristics of COVID-19 vaccine/vaccination** | **Vaccine A** | **Vaccine B** |
| --- | --- | --- |
| **Severe side effects that may require you to be hospitalized** | 1 in 100, 000 will have severe side effects | 1 in 100 will have severe side effects |
| **Effectiveness** | **70-90%** | **Less than 70%** |
| **Content** | - **Halal certified** - **Contains** ingredients from cow (bovine) | - **Non-Halal certified** - **Contains** ingredients from cow (bovine) |
| **Schedule (all COVID-19 vaccinations are free)** | During **office hours (weekdays, 8am-5pm)** in the **private** healthcare facilities | During **office hours (weekdays, 8am-5pm)** in the **public** healthcare facilities |
| **Distance from home to vaccination facilities** | **10 km** | **1 km** |
| **I choose**  (please tick one box only) | **Vaccine A**  **Vaccine B**  **No vaccination** | |

**Scenario 5 of 11:** Based on the vaccine / vaccination characteristics below, what is your choice?

| **Characteristics of COVID-19 vaccine/vaccination** | **Vaccine A** | **Vaccine B** |
| --- | --- | --- |
| **Severe side effects that may require you to be hospitalized** | 1 in 1,000 will have severe side effects | 1 in 10,000 will have severe side effects |
| **Effectiveness** | **Less than 70%** | **Above 90%** |
| **Content** | - **Non-Halal certified** - **No** ingredients from cow (bovine) | - **Halal certified** - **Contains** ingredients from cow (bovine) |
| **Schedule (all COVID-19 vaccinations are free)** | **Out-of-office hours (after 5pm, weekends/public holidays)** in the **private** healthcare facilities | **Out-of-office hours (after 5pm, weekends/public holidays)** in the **public** healthcare facilities |
| **Distance from home to vaccination facilities** | **5 km** | **10 km** |
| **I choose**  (please tick one box only) | **Vaccine A**  **Vaccine B**  **No vaccination** | |

**Scenario 6 of 11:** Based on the vaccine / vaccination characteristics below, what is your choice?

| **Characteristics of COVID-19 vaccine/vaccination** | **Vaccine A** | **Vaccine B** |
| --- | --- | --- |
| **Severe side effects that may require you to be hospitalized** | 1 in 100,000 will have severe side effects | 1 in 1,000 will have severe side effects |
| **Effectiveness** | **70-90%** | **Less than 70%** |
| **Content** | - **Halal certified** - **Contains** ingredients from cow (bovine) | - **Non-Halal certified** - **Contains** ingredients from cow (bovine) |
| **Schedule (all COVID-19 vaccinations are free)** | **Out-of-office hours (after 5pm, weekends/public holidays)** in the **public** healthcare facilities | **Out-of-office hours (after 5pm, weekends/public holidays)** in the **private** healthcare facilities |
| **Distance from home to vaccination facilities** | **1 km** | **50 km** |
| **I choose**  (please tick one box only) | **Vaccine A**  **Vaccine B**  **No vaccination** | |

**Scenario 7 of 11:** Based on the vaccine / vaccination characteristics below, what is your choice?

| **Characteristics of COVID-19 vaccine/vaccination** | **Vaccine A** | **Vaccine B** |
| --- | --- | --- |
| **Severe side effects that may require you to be hospitalized** | 1 in 100 will have severe side effects | 1 in 10,000 will have severe side effects |
| **Effectiveness** | **Above 90%** | **70-90%** |
| **Content** | - **Non-Halal certified** - **Contains** ingredients from cow (bovine) | - **Halal certified** - **No** ingredients from cow (bovine) |
| **Schedule (all COVID-19 vaccinations are free)** | During **office hours (weekdays, 8am-5pm)** in the **public** healthcare facilities | **Out-of-office hours (after 5pm, weekends/public holidays)** in the **private** healthcare facilities |
| **Distance from home to vaccination facilities** | **50 km** | **1 km** |
| **I choose**  (please tick one box only) | **Vaccine A**  **Vaccine B**  **No vaccination** | |

**Scenario 8 of 11:** Based on the vaccine / vaccination characteristics below, what is your choice?

| **Characteristics of COVID-19 vaccine/vaccination** | **Vaccine A** | **Vaccine B** |
| --- | --- | --- |
| **Severe side effects that may require you to be hospitalized** | 1 in 100 will have severe side effects | 1 in 100,000 will have severe side effects |
| **Effectiveness** | **70-90%** | **Above 90%** |
| **Content** | - **Halal certified** - **Contains** ingredients from cow (bovine) | - **Non-Halal certified** - **No** ingredients from cow (bovine) |
| **Schedule (all COVID-19 vaccinations are free)** | During **office hours (weekdays, 8am-5pm)** in the **private** healthcare facilities. | **Out-of-office hours (after 5pm, weekends/public holidays)** in the **private** healthcare facilities |
| **Distance from home to vaccination facilities** | **50 km** | **5 km** |
| **I choose**  (please tick one box only) | **Vaccine A**  **Vaccine B**  **No vaccination** | |

**Scenario 9 of 11:** Based on the vaccine / vaccination characteristics below, what is your choice?

| **Characteristics of COVID-19 vaccine/vaccination** | **Vaccine A** | **Vaccine B** |
| --- | --- | --- |
| **Severe side effects that may require you to be hospitalized** | 1 in 1,000 will have severe side effects | 1 in 100,000 will have severe side effects |
| **Effectiveness** | **Above 90%** | **70-90%** |
| **Content** | - **Non-Halal certified** - **Contains** ingredients from cow (bovine) | - **Halal certified** - **Contains** ingredients from cow (bovine) |
| **Schedule (all COVID-19 vaccinations are free)** | During **office hours (weekdays, 8am-5pm)** in the **private** healthcare facilities | **Out-of-office hours (after 5pm, weekends/public holidays)** in the **public** healthcare facilities |
| **Distance from home to vaccination facilities** | **5 km** | **10 km** |
| **I choose**  (please tick one box only) | **Vaccine A**  **Vaccine B**  **No vaccination** | |

**Scenario 10 of 11:** Based on the vaccine / vaccination characteristics below, what is your choice?

| **Characteristics of COVID-19 vaccine/vaccination** | **Vaccine A** | **Vaccine B** |
| --- | --- | --- |
| **Severe side effects that may require you to be hospitalized** | 1 in 100,000 will have severe side effects | 1 in 10,000 will have severe side effects |
| **Effectiveness** | **Above 90%** | **Less than 70%** |
| **Content** | - **Non-Halal certified** - **No** ingredients from cow (bovine) | - **Halal certified** - **Contains** ingredients from cow (bovine) |
| **Schedule (all COVID-19 vaccinations are free)** | **Out-of-office hours (after 5pm, weekends/public holidays)** in the **public** healthcare facilities | During **office hours (weekdays, 8am-5pm)** in the **private** healthcare facilities |
| **Distance from home to vaccination facilities** | **10 km** | **5 km** |
| **I choose**  (please tick one box only) | **Vaccine A**  **Vaccine B**  **No vaccination** | |

**Scenario 11 of 11:** Based on the vaccine / vaccination characteristics below, what is your choice?

| **Characteristics of COVID-19 vaccine/vaccination** | **Vaccine A** | **Vaccine B** |
| --- | --- | --- |
| **Severe side effects that may require you to be hospitalized** | 1 in 100,000 will have severe side effects | 1 in 100 will have severe side effects |
| **Effectiveness** | **Above 90%** | **Less than 70%** |
| **Content** | - **Halal certified** - **No** ingredients from cow (bovine) | - **Non-Halal certified** - **Contains** ingredients from cow (bovine) |
| **Distance from home to vaccination facilities** | **1 km** | **50 km** |
| **I choose**  (please tick one box only) | **Vaccine A**  **Vaccine B**  **No vaccination** | |

**Section 2: Demographic**

| Q1. | Sex | Male Female |
| --- | --- | --- |
| Q2. | Ethnicity | Malay  Chinese  Indian  Semenanjung Aborigines  Sabah Native  Sarawak Native  Others : ____________________________ |
| Q3. | Current state of residence | Johor  Kedah  Kelantan  Malacca  Negeri Sembilan  Pahang  Penang  Perak  Perlis  Sabah  Sarawak  Selangor  Terengganu  Federal Territory Kuala Lumpur  Federal Territory Labuan  Federal Territory Putrajaya |
| Q4. | Highest Education Level | No Formal Education  Primary Education  Secondary education (up to form 5)  Form 6 / certificate / diploma / A-level / Pre-university course  Tertiary education (degree, master, PhD, DrPH) |
| Q5. | Employment status | Private sector employee  Government sector employee  Self employed  Unemployed  Student  Retiree/Pensioner |
| Q6. | Marital status | Single  Married  Separated/Divorced  Widow/Widower  Living with partner |
| Q7. | Are you currently pregnant? | Yes No |
| Q8. | Do you live with elderly (60 years old and above) *and/or* children *and/or* pregnant women *and/or* person with existing health conditions?  If **“YES”** tick all options that apply | Yes No    Elderly  Children  Pregnant Women  Person with existing health conditions |
| Q9. | Total **Monthly Household** Income  *(Definition: A household consists of persons who live together and make common provision for food and other living essentials.)* | < RM 2500  RM 2500 – RM 3169  RM 3170 – RM 3969  RM 3970 – RM 4849  RM 4850 – RM 5879  RM 5880 – RM 7099  RM 7100 – RM 8699  RM 8700– RM 10959  RM 10960 – RM 15039  >RM 15039 |
| Q10. | Are you a member of any of the following populations?  ***Tick all that applies** | Healthcare workers  Non-healthcare essential workers (e.g., army, police, provision of food and essential goods, utility workers)  Stay in crowded living household  Difficult to maintain physical distancing at workplace  **None** of the above |
| Q11. | Have you been infected with the COVID-19? | Yes No |
| Q12. | Do you have any existing health condition?  ***Tick all that applies** | No existing health conditions  Diabetes  High blood pressure  High cholesterol  Heart Disease  Stroke  Respiratory disease  Liver diseases  Kidney diseases  Allergic conditions  Autoimmune disease  Cancer  Others, please specify:  ____________________________________________ |
| Q13. | What is the mode of transport that you usually use to seek treatment at a healthcare facility? | Own vehicle  Public transport (e.g., buses, light rail transit (LRT), trains etc)  Taxis, e-hailing (e.g., Grab)  Transport by Family/Relatives/Friends  Walking |
| Q14. | Do you have *MySejahtera application* account? | Yes No  If **“YES”**, do you use it (e.g. for checking in/checking daily COVID-19 statistics)  Yes No |
| Q15. | If you were given a choice to be vaccinated for COVID-19, would you take it? | Yes No  If **“YES”**, which one would you choose? *(You may choose* ***more than one****)*  Pfizer  AstraZeneca  Sinovac Biotech  CanSino Biologics  Gamaleya Research Institute (Sputnik-V)  Sinopharm  Moderna  No specific preference |

**Section 3: Other Relevant Questions**

**COVID-19 Risk Perception (2 Questions)**

1. In your opinion, how risky are you to get infected with COVID-19 in the next 6 months?

Very Low

Low

Medium

High

Very High

1. If you were infected with COVID-19, how serious would it affect your health?

Very Mild

Mild

Moderate

Serious

Very Serious

**General views on vaccines/vaccination (5 Questions)**

1. Do you believe that vaccines can protect you from serious diseases (e.g., measles, polio, smallpox, Tuberculosis etc)?

Yes

No

1. Have you ever been reluctant to get a vaccination in the past?

Yes

No

1. Have you ever refused a vaccination in the past?

Yes

No

1. Would you encourage your immediate family members (children, spouses etc.) to be vaccinated for COVID-19?

Yes

No

1. Would you encourage your friends to be vaccinated for COVID-19?

Yes

No

**Efforts against the COVID-19 pandemic (4 Questions)**

1. Overall, I trust the Government for the management of COVID-19.

Strongly Disagree

Disagree

Not Sure

Agree

Strongly Agree

1. The government takes into consideration of experts' advice (e.g., epidemiologists, virologists, paediatricians, psychologists, etc.) to manage the current COVID-19 situation.

Strongly Disagree

Disagree

Not Sure

Agree

Strongly Agree

1. The government is allocating enough resources to enforce the adopted measures and guidelines to manage the current COVID-19 situation.

Strongly Disagree

Disagree

Not Sure

Agree

Strongly Agree

1. In general, how reliable do you think the following sources of information are in relation to the COVID-19 pandemic?

| **No.** | **Information** | **Strongly Not Reliable** | **Not Reliable** | **Not Sure** | **Reliable** | **Strongly Reliable** |
| --- | --- | --- | --- | --- | --- | --- |
| Q1. | Media (TV, newspapers, radio, etc.) |  |  |  |  |  |
| Q2. | Official government websites (e.g., Ministry of Health, National Government) |  |  |  |  |  |
| Q3. | Social media platform (e.g., WhatsApp, Facebook) |  |  |  |  |  |
| Q4. | Doctors |  |  |  |  |  |
| Q5. | Friends, relatives and acquaintances |  |  |  |  |  |
| Q6. | Scientists |  |  |  |  |  |

Thank you for your responses!

Your participation is much appreciated.
